# Gastric peroral endoscopic myotomy mucosotomy closure using a novel hand suturing device

**DOI:** 10.1055/a-2334-2750

**Published:** 2024-06-21

**Authors:** Pedro J. Rosón, Francisco Fernandez Cano, Mari A. Romero, Alicia Paris

**Affiliations:** 1 Digestive System and Endoscopy Unit, Hospital Vithas Xanit, Benalmadena, Spain

A 37-year-old patient with clinical signs of gastroparesis underwent a gastric peroral endoscopic pyloromyotomy (G-POEM) technique. After creating the submucosal tunnel and sectioning the pylorus, it was necessary to suture the gastric mucosotomy. Generally, this is done with standard endoscopic clips, but given that the gastric mucosa and muscular mucosa are thicker than the esophageal mucosa, it is sometimes difficult to close the mucosotomy, and in other cases early dehiscence of the closure has been described, requiring a second endoscopic closure 1 .

Recently, a new endoscopic suturing system has been introduced, consisting of a flexible needle holder, which allows standard surgical needles to be manipulated to perform continuous manual suturing ( Fig. 1 ).

In our case ( Video 1 ), after performing the standard G-POEM technique, endoscopic suturing was performed manually to minimize the risk of dehiscence and facilitate the suturing technique. A 3–0 barbed suture was used, as this type of suture does not require a knot to be tied to secure it. With only three stiches ( Fig. 2 **a** ), we managed to completely close the mucosal incision; a final fourth stitch was applied in the opposite direction and secured with a simple knot to complete the closure ( Fig. 2 **b** ).

Pyloromyotomy and posterior closure of the mucosotomy using the manual suture device.Video 1

**Fig. 1:**
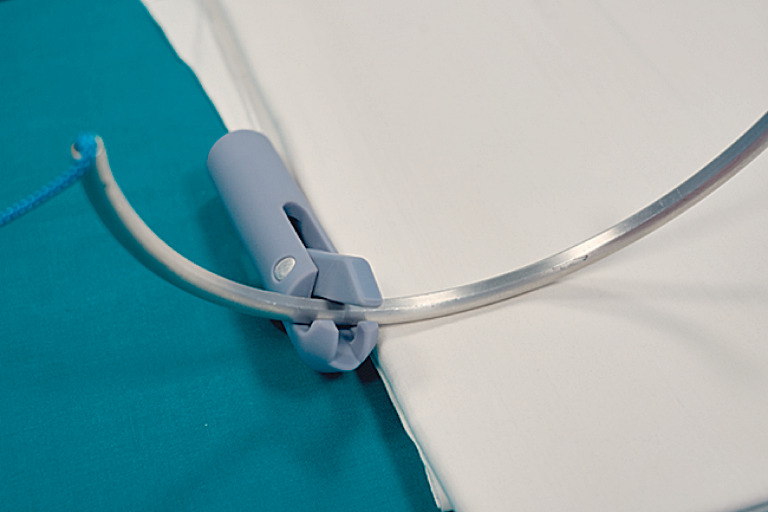
Suturing device with surgical needle.

**Fig. 2:**
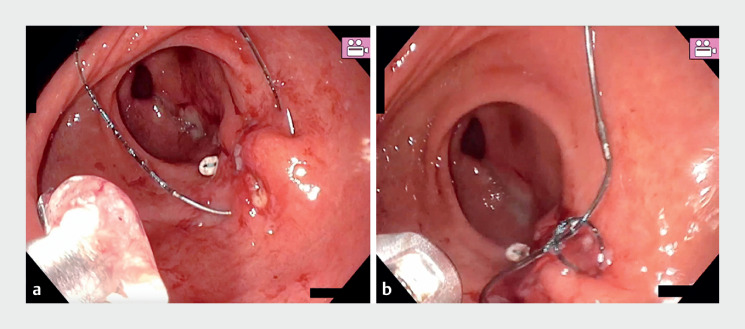
**a** Needle passing through both edges of the mucosotomy. **b** The final knot to secure closure.

This recently introduced endoscopic hand suturing method for closure of mucosal resection defects can be used for the closure of mucosotomies in third space techniques. We believe that the development of new suturing techniques, such as the one described here, will facilitate and provide security to the closures of third space techniques in more complex locations, such as in the presented case.

Endoscopy_UCTN_Code_TTT_1AO_2AO

## Notes

Endoscopy E-Videos https://eref.thieme.de/e-videos
*E-Videos* is an open access online section of the journal *Endoscopy* , reporting on interesting cases and new techniques in gastroenterological endoscopy. All papers include a high-quality video and are published with a Creative Commons CC-BY license. Endoscopy E-Videos qualify for HINARI discounts and waivers and eligibility is automatically checked during the submission process. We grant 100% waivers to articles whose corresponding authors are based in Group A countries and 50% waivers to those who are based in Group B countries as classified by Research4Life (see: https://www.research4life.org/access/eligibility/ ). This section has its own submission website at https://mc.manuscriptcentral.com/e-videos .

## References

[1] Mekaroonkamol P Shah R Cai Q Outcomes of per oral endoscopic pyloromyotomy in gastroparesis worldwide World J Gastroenterol 2019 25 909 922 10.3748/wjg.v25.i8.909 30833798 PMC6397720

